# Faculty of the Medical Department University of Texas

**Published:** 1891-09

**Authors:** 


### EDITORIAL DEPARTMENT.

F. E. DANIEL, M. D., EDITOR AND PUBLISHER.

THIS JOURNAL, by unanimous vote of the members, was made the Official Organ of the Austin District Medical Society.

COLLABORATORS:

*Dr. R. M. Swearingen, Austin.*
*Prof. B. E. Hadra, M. D., Galveston.*
*Prof. Geo. Cupples, M. D., San Antonio.*
*Dr. T. C. Osborn, Cleburne, Texas.*
*Dr E. J. Doering, Chicago.*
*Dr. E. J. Beall, Fort Worth.*
*Dr Odo Betz, Germany.*
*Prof. W. B. Rogers, M. D., Memphis.*
*Dr. J. W. McLaughlin, Austin.*
*Dr. T. J. Tyner, Austin.*
*Prof. J. F. Y. Paine, M. D., Galveston.*
*Dr. R. H. L. Bibb, Mexico.*
*Dr. T. J. Bennett. Austin.*
*Dr. Bat Smith, Wharton.*
*Dr. E. Meierhof, New York.*
*L. H. Luce, M. D., West Tisbury, Mass.*

### THE FACULTY OF THE MEDICAL DEPARTMENT TEXAS UNIVERSITY.

In our August issue, just as the last form was closed, it was learned and announced that "Dr. Finney, of Baltimore, had been elected to the Chair of Surgery,"—the Board of Regents being at that hour in session in Galveston, wrestling with several scores of applications for the several chairs. Next mail brought us full particulars. The newly chosen Faculty consists of the following:

Chair of Surgery—Dr. J. T. M. Finney, late out-door assistant to the Johns-Hopkins Hospital; salary, $3000.

Chair of Obstetrics and Gynecology—Dr. J. F. Y. Paine, (Dean of the Faculty), already announced; salary, $2500.

Chair of Practice of Medicine—Dr. H. A. West, already announced; salary, $2500.

Chair of Physiology and Hygiene—Dr. A. G. Clopton, of Jefferson, Texas; salary, $3000.

Chair of Anatomy—Dr. Wm. Keiller, of Edinburgh, Eng.; salary, $2500.

Chair of Bacteriology and Histology—Dr. A. J. Smith, of Philadelphia; salary, $3000.

Chair of Chemistry and Toxology—Dr. Seth M. Morris, of Austin; salary, $2000.

Chair of Materia Medica—Dr. Edw. Randall; salary, $2500.

Demonstrator of Anatomy—Dr. Geo. H. Lee, of Galveston.

We give below a brief sketch of the Professor of Chemistry, believing it will be read with unusual interest, inasmuch as he is a native Texan, and one of the very first crop of our University graduates. It is a matter of much pride and gratification that Texas has a University capable of turning out young men who are able to fill such positions, and it is creditable to the Board of Regents that they recognized the claims of one of their own graduates; it is a fitting reward for long and earnest application, whereby this young man qualified himself, and shows that such endeavors are fully appreciated. This should stimulate all young men to emulate his example; it shows also that the prize is within the reach of those who determine to succeed, and know no such word as fail.

SETH MABRY MORRIS, B.S., M.D., Prof. Chemistry Med. Dept. University of Texas, was born in Austin in 1867. His parents are Dr. W. A. Morris and Lucinda Mabry Morris. He received his preliminary education in the schools of Austin, and on completion of the University of Texas, matriculated amongst the very first pupils. He took the five year's course, devoting special attention to chemistry under Profs. Mallett and Everhart, and Physics under Dr. Macfarlane. In both these branches he won distinction; and during his last senior year he was chosen by Professor Everhart as laboratory assistant. Graduating at the University in 1888, with the degree of B. S., he at once began the study of medicine in his father's office, and in the fall of that year entered the College of Physicians and Surgeons in New York. Here he took the required three years course, giving special attention to chemistry under the instruction of Prof. Chandler, and graduated M. D. from that school in 1891. In addition to the degree M. D. conferred upon him, he was awarded a "special examination diploma" and a cash prize, being one of the ten to whom special honors were awarded, in a graduating class of one hundred and fifty odd.

Dr. Morris is a man of studious and exemplary habits, and is a member of the Christian church. His friends predict for him a brilliant career.

The biography of Professor Paine has already appeared in this JOURNAL—in connection with his Presidency of the Texas State Medical Association. We have no data of Dr. West and the other members.

Dr. West is Secretary of the Texas State Medical Association, and was Professor of Practice in the late Texas Medical College.

Dr. Clopton, the Professor of Physiology, is well known to the Texas profession with whom he is a prime favorite.

Dr. Edw. Randall is the son of the late Dr. E. Randall, of Galveston, one of the pioneer physicians of that city, and is also well known and popular.

Dr. George H. Lee, the Demonstrator is well and favorably known, and filled the Chair of Anatomy in the late Texas Medical College.

Dr. Wm. Keiller is a F. R. C. S., and bears a number of other titles; among them the very (to Americans) remarkable one of "honorary chloroformist." He comes endorsed by the leading anatomists and surgeons of England as a most accomplished anatomist; though there are those who think that it was unnecessary to go to England or Scotland for an anatomist.

Dr. Finney, the Surgeon-elect, also comes highly recommended. We know nothing of him further than that we see his name in the bulletin of the Johns-Hopkins Hospital as "out-door assistant to Dr. Halsted."

